# An Integrated Molecular Database on Indian Insects

**DOI:** 10.6026/97320630014042

**Published:** 2018-02-28

**Authors:** Maria Pratheepa, Thiruvengadam Venkatesan, Gandhi Gracy, Sushil Kumar Jalali, Rajagopal Rangheswaran, Jomin Cruz Antony, Anil Rai

**Affiliations:** 1 ICAR-National Bureau of Agricultural Insect Resources, H.A. Farm Post, P.Bag No: 2491, Bellary Road, Hebbal, Bengaluru - 560 024. India; 2 Department of Computer Science, Jain University, Bengaluru - 560 027, India; 3 ICAR-Indian Agricultural Statistical, Research Institute, New Delhi - 110 012, India

**Keywords:** molecular database, insect, agriculture

## Abstract

MOlecular Database on Indian Insects (MODII) is an online database linking several databases like Insect Pest Info, Insect Barcode Information System (IBIn), Insect Whole Genome sequence, Other Genomic Resources of National Bureau of Agricultural Insect Resources (NBAIR), Whole Genome sequencing of Honey bee viruses, Insecticide resistance gene database and Genomic tools. This database was developed with a holistic approach for collecting information about phenomic and genomic information of agriculturally important insects. This insect resource database is available online for free at http://cib.res.in.

**Availability:**

http://cib.res.in/

## Background

**Figure 1:**
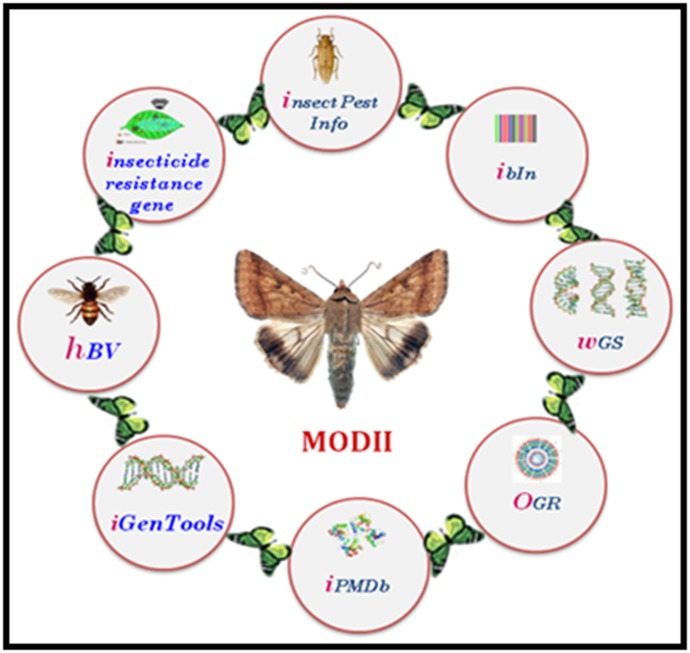
MOlecular Database on Indian Insects (MODII)

Insects play a major role in agricultural ecosystems [1] because there are beneficial insects as well as pests. Insect pests cause damage in crop production. In India the crop loss was around 8,63,884 million rupees due to insect pests [2]. The crop production loss is around 15-25% due to insect pests, weeds and diseases [3]. There is a need for integrated, up-to-date collection of phenomics and genomics information of agriculturally important insects, which can serve as reference for the entomologists especially for pest management. Hence, effort has been made for the development of MOlecular Database on Indian Insects (MODII) which is online database contains several databases like Insect Pest Info, Insect Barcode Information System (IBIn), Insect Whole Genome sequencing (WGS), Other Genomic Resources (OGR) of National Bureau of Agricultural Insect Resources (NBAIR), Whole Genome sequencing of Honeybee viruses (hBV), Insecticide resistance gene database (IRGD) and Genomic tools (iGenTools). MODII has been developed based on three-tier architecture of the client-server technology. This database developed with the holistic approach, which gives information about phenomic and genomic information of agriculturally important insects. This database gives sequence information collected from the National Centre for Biotechnology Information (NCBI) [4], the sequences from Division of Genomic Resources, Indian Council of Agricultural Research (ICAR) - National Bureau of Agricultural Insect Resources (NBAIR), Bengaluru, India and other public domain. This database is available as online at http://cib.res.in in the local server of ICARNBAIR and updated regularly. The biological database in agriculture has been designed and the sequence information is available in the local server of ICAR-Indian Agricultural Statistics Research Institute [5]. The entomologists who involved in molecular research can use this information for their research work. Different databases of MODII have been given in Figure 1 and the brief description of MODII is explained in this paper.

## Methodology

**Figure 2:**
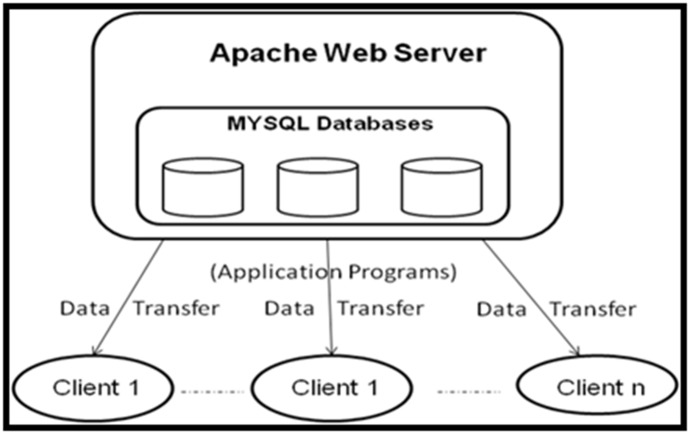
Diagram of three-tier architecture of MOlecular Database on Indian Insects

**Figure 3:**
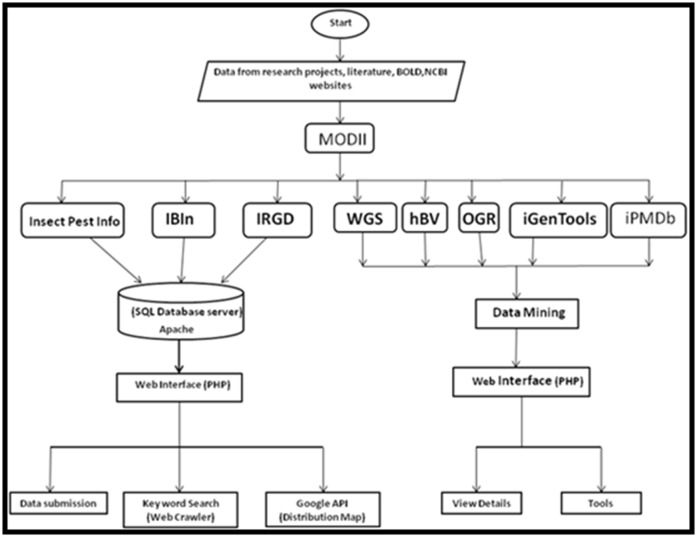
Block diagram of Molecular Database on Indian Insects

Molecular Database On Indian Insects (MODII) has been developed based on three-tier architecture of the client-server technology and multiple users can access at a time. MYSQL has been used to store the information with Apache 2 web server as an interface in Linux environment. PHP has been used for developing programs for login facility, submission of data in the form of sequences, insect information, etc. Google API, Web crawler technique, Java applets have been used for other features like 'Keyword Search', 'View information' and for Distribution maps. The three-tier architecture of MODII has been depicted in Figure 2. MODII contains several databases and tools and the block diagram of MODII is given in Figure 3.

### Insect Pest Info

**Figure 4:**
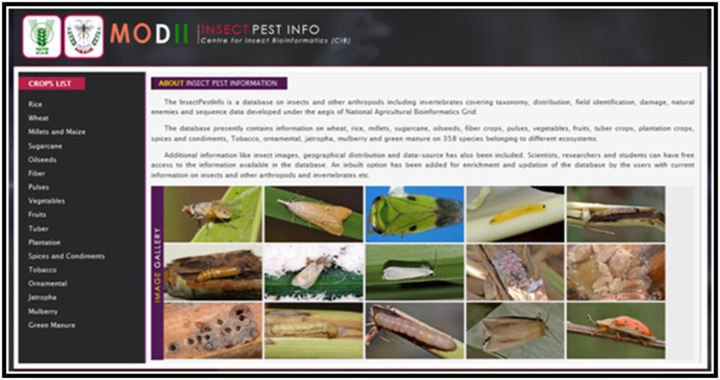
Homepage of Insect Pest Info database

The 'Insect Pest Info' database has been developed to furnish information on insect pests based on the crop selection. The database gives information about the common name of the pest, the scientific name of the pest, taxonomy, identification and damage details on the crop, distribution map and natural enemies with QR code. There are around totally 400 pest details in the database. The crops list contains Rice, Wheat, Millets and Maize, Sugarcane, Oilseeds, Fiber, Pulses, Vegetables, Fruits, Plantation, Spices and Condiments, Tobacco, Ornamental, Jatropha and Green manure. The web pages have been developed for the pests of these crops and the database has been updated regularly. The homepage of Insect Pest Info has been given in Figure 4.

### Insect Barcode Informatica

The database for generation of DNA barcoding of insects named Insect Barcode Informatica (IBIn) was developed, which contains the information on different orders of insects, viz., Coleoptera, Diptera, Embioptera, Ephemeroptera, Hemiptera, Hymenoptera, Lepidoptera, Neuroptera, Odonata, Orthoptera, Thysanoptera and Trichoptera. At present IBIn database contains 804 insect details with nucleotide sequences and barcodes. In India, only 1274 insect species have been barcoded and in world 127694 insect species have been barcoded even though the insect population is more than 1 million worldwide [6]. IBIn provides the statistical data about the number of species DNA barcoded in the World and in India. Submitting the nucleotide sequence at http://www.cib.res.in/ibin/create-barcode.php can generate DNA barcode. Researchers in India can register and submit the nucleotide sequences to IBIn for generation of barcode and for storage of sequence information. The detailed information about IBIn has been already published [7].

### Database on Whole Genome Sequencing (WGS) of important insects

**Figure 5:**
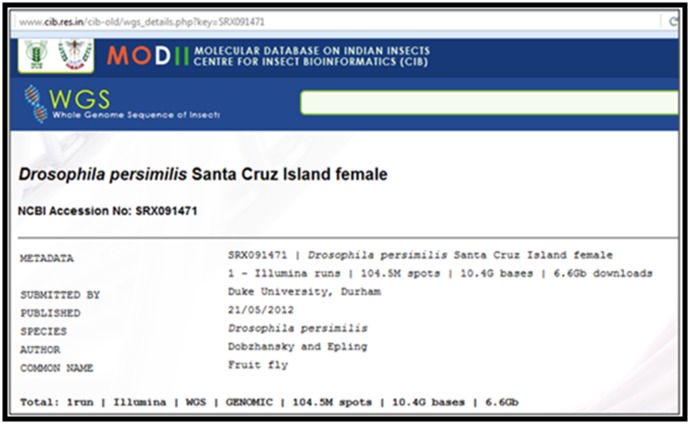
Metadata of Drosophila persimilis Santa Cruz Island female

Developed Whole Genome Sequencing (WGS) database along with metadata and links have been established for 20 WGS of agriculturally important insects of different orders like Coleoptera, Diptera, Hemiptera, Hymenoptera and Lepidoptera to NCBI website. The list of insects has been given in supplementary Table 1. The metadata contains Submitted by, Date of Publication, NCBI accession number and Common name of the insect. The screen shot of the metadata of Drosophila persimilis Santa Cruz Island female has been given in Figure 5.

### Other Genomic Resources (OGR)

Other Genomic Resources (OGR) has been developed for microbial for which genome sequencing has been done from the institute ICAR-NBAIR. Presently, it contains 203 accessions along with metadata. Links have been established for these accessions to the NCBI website. The metadata contains Meta-Info, Voucher- Info, Organism, Classification and Authors.

### Honeybee Viruses Genome

Honeybee viruses are causing problem in honeybee production [8]. This database hosts the complete genomic information on honeybee viruses, which infects different species and populations of honeybees in India. This is an important database, which is initiative in the Honeybee viral diseases identification and management. Presently, this database contains 7 Whole Genome Sequence of Sacbrood virus from ICAR-NBAIR (JX194121, JX270795, JX270796, JX270797, JX270798, JX270799 and JX270800), along with the metadata and Whole Genome Sequences of Acute bee paralysis virus, Black queen cell virus, Deformed wing virus, Kashmir bee virus, Sacbrood virus and Thai Sacbrood viruses.

### Insecticide Resistance Gene Database

**Figure 6:**
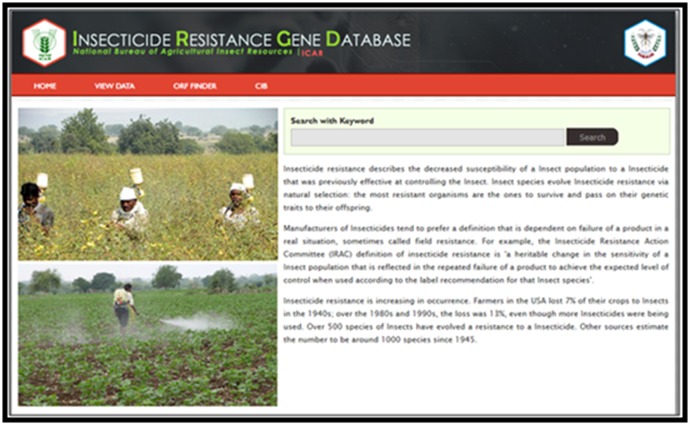
Homepage of Insecticide Resistance Gene Database (IRGD)

Managing of insect pests is a challenge now-a-days since agricultural pests are developing resistance against insecticides like organophosphates, synthetic pyrethroids, organo chlorinates and other new groups [9]. Insecticide resistance is a widespread phenomenon and leads to frequent and overuse of pesticides that pose a risk to the environment and human health. Insecticide resistance gene database (IRGD) for important pests is essential to carry out molecular studies on insecticide resistant genes like Cytochrome P450, Acetylcholinesterase (AchE), Knock down resistance (KDR) and Resistant to dieldrin (Rdl) gene. Hence, Insecticide Resistant Gene Database (IRGD) has been developed and this database helps researchers in designing novel molecules for overcoming insecticide resistance in agricultural pests. Presently, IRGD contains 851 sequences for the pests Aphis gossypii Glover, Acyrthosiphon pisum Harris, Bemisia tabaci Gennadius, Helicoverpa armigera Hubner, Plutella xylostella Linnaeus, Spodoptera exigua Hubner, Spodoptera litura Fabricius, Nilaparvata lugens Stal, Myzus persicae Sulzer, Tribolium castaneum Herbst and Lucinodes orbonalis Guenee with key features like Search, View, ORF Finder, etc. and this database is updated regularly. The homepage of the IRGD database is given in Figure 6.

### iPMDb

Insect Protein Model Database is under progress, which gives the 3-D structure of insect protein prediction models. This helps to understand the insect protein structures, the target site for the insecticides and the mutations in these proteins caused the resistance towards insecticides.

### iGenTools

Genomic tools are necessary to carry out analysis on the sequence data and hence some of the tools like calculation of GC and AT percentage, DNA to protein sequence (translation), reverse compliment, protein parameter analysis tool have been developed and included into MODII.

## Conclusion

MOlecular Database on Indian Insects (MODII) contains several databases like Insect Pest Info, Insect Barcode Information System (IBIn), Insect Whole Genome sequence (WGS), Other Genomic Resources (OGR) of National Bureau of Agricultural Insect Resources (NBAIR), Whole Genome sequencing of Honey bee viruses (hBV), Insecticide resistance gene database (IRGD) and Genomic tools (iGenTools). Molecular Database on Indian Insects (MODII) is available for free at http://cib.res.in. Phenomic and genomic information of agriculturally important insects of India can be accessed in one platform through this database. The insect resource database is useful for farmers, students, entomologists, and researchers to get information on agriculturally important insects.

## Figures and Tables

**Table 1:** List of insects included in WGS database

S.No	Insect Order	Name of the insect
1	Coleoptera	*Dendroctonus ponderosae Hopkins*
2	Hemiptera	*Acyrthosiphon pisum* Harris
3	Hymenoptera	*Apis mellifera* Linnaeus
4		*Camponotus floridanus* Buckley
5		*Herpegnathos saltator* T. C. Jerdon,
6	Lepidoptera	*Bombyx mori* Linnaeus
7		*Heliconius melpomene* Linnaeus
8	Diptera	*Drosophila ananassae*
9		*Drosophila biarmipes*
10		*Drosophila bipectinata*
11		*Drosophila elegans*
12		*Drosophila eugracilis*
13		*Drosophila ficusphila*
14		*Drosophila kikkawai*
15		*Drosophila melanogaster*
16		*Drosophila persimilis*
17		*Drosophila pseudoobscura*
18		*Drosophila rhopaloa*
19		*Drosophila takahashi*
20		*Drosophila yakuba*
